# Blood molecular subtypes to guide precision treatment strategies in systemic juvenile idiopathic arthritis

**DOI:** 10.1186/s13075-025-03498-8

**Published:** 2025-02-08

**Authors:** In-Woon Baek, Jung Woo Rhim, Kyung-Su Park, Ki-Jo Kim

**Affiliations:** 1 Division of Rheumatology, Department of Internal Medicine, College of Medicine, Ewha Womans University, Seoul, Republic of Korea; 2 Department of Pediatrics, Daejeon St. Mary’s Hospital, College of Medicine, The Catholic University of Korea, Seoul, Republic of Korea; 3 Division of Rheumatology, Department of Internal Medicine, St. Vincent’s Hospital, College of Medicine, The Catholic University of Korea, Seoul, Republic of Korea; 4 St. Vincent’s Hospital, 93 Jungbu-daero, Paldal-gu, Suwon, Gyeonggi-do 16247 Republic of Korea

**Keywords:** Systemic juvenile idiopathic arthritis, Molecular subtype, Neutrophil, Interleukin-1

## Abstract

**Background:**

Systemic juvenile idiopathic arthritis (sJIA) is the most severe subtype of JIA, with a combination of diverse clinical manifestations and a variable clinical course. A comprehensive understanding of molecular signatures at the systems level and the discovery of molecular subtypes are the initial steps toward personalized medicine in sJIA.

**Methods:**

A blood transcriptomic dataset was collected from patients with systemic JIA (sJIA) (*n* = 168), polyarticular JIA (*n* = 254), oligoarticular JIA (*n* = 96), enthesitis-related arthritis (*n* = 40), and healthy controls (*n* = 220). Gene expression profiles were filtered for differentially expressed genes and unsupervised clustering, gene set enrichment, and network-based centrality analyses. The molecular signatures of three novel sJIA subgroups (designated as C1, C2, and C3) were investigated, focusing on their distinct features and treatment responses.

**Results:**

Neutrophil degranulation and the IL-1 signaling pathway were the shared key processes for the three subgroups. Proinflammatory signals, including TNF, IL-6, TLR, and G-CSF signaling pathways, were identified with variation across the subgroups. C1 was the most inflammatory subset with a high-risk profile for macrophage activation syndrome. The C2 subset had the most activated IL-1 and IL-18 signaling pathways. C2 and C3 have higher levels of interferon-stimulated signatures. In a canakinumab-treated dataset, treatment response was correlated with IL1B expression and NF-κB signaling pathway, and neutrophil activation-associated processes were effectively suppressed in a good responder group. GSK3B and p38 MAPK inhibitors showed a significant counteracting effect on the perturbed gene expression of sJIA.

**Conclusions:**

Neutrophil activation was the key feature in active sJIA. The three molecular subtype scheme enables the formulation of precision medicine strategies in sJIA.

**Supplementary Information:**

The online version contains supplementary material available at 10.1186/s13075-025-03498-8.

## Introduction

Systemic juvenile idiopathic arthritis (sJIA) is the most severe subtype of juvenile idiopathic arthritis (JIA) and is characterized by chronic arthritis, intermittent high-spiking fever, maculopapular rash, hepatosplenomegaly, lymphadenopathy, and elevated levels of acute-phase reactants [1, 2]. sJIA account for approximately 10% of patients with JIA and macrophage activation syndrome (MAS) occurs at disease onset and/or during the disease course in approximately 10% of patients [1, 2].

Traditionally, non-steroidal anti-inflammatory drugs (NSAIDs) and glucocorticoids were the mainstay in the treatment of sJIA. However, the introduction of several targeted drugs against interleukin (IL)-1 and IL-6 has substantially improved the treatment outcome over the past two decades. Nearly 50% of patients achieve remission with glucocorticoid discontinuation, and up to two-thirds showed minimal disease activity [1]. Despite these dramatic responses, approximately 15–20% of patients still have no response to single-drug biologic therapy and approximately 40% continued to have active disease while on single biologic therapy [3]. In a study using registry data, 24% of patients continued to have chronically uncontrolled sJIA despite exposure to ≥ 2 biologic disease-modifying antirheumatic drugs (DMARDs) [4].

The development of sJIA is driven by the activation of the innate immune response and critically mediated by key cytokines such as IL-1, IL-6, and IL-18 [1, 5]. However, the clinical course of sJIA is fairly variable depending on episodic flares of systemic and/or arthritic features, and MAS, the most devastating complication, develops only in a small subset of patients [1, 6, 7]. Evidence from biological and therapeutic studies suggests different cytokine patterns across patients and the existence of subgroups with different characteristics toward autoinflammatory or autoimmune responses [8, 9]. This indicates that molecular and cellular heterogeneity lies behind the diversity in the clinical course and therapeutic outcome.

Integrative modular analysis of blood molecular signatures has greatly enhanced our understanding of key pathogenic pathways in the clinical features and the divergent and shared molecular characteristics of disease subgroups in autoimmune rheumatic disease such as systemic lupus erythematosus, Sjögren’s syndrome, and rheumatoid arthritis [10–15]. In addition, a combination of multiple datasets could increase the chance of systematically clustering the samples and detecting the significant differences between subgroups while reducing sampling bias. In the present study, we used biomedical data libraries to collect the blood cell transcriptomic datasets from patients with JIA, including sJIA, polyarticular JIA (paJIA), oligoarticular JIA (oaJIA), and enthesitis-related arthritis (ERA), and identified the shared and distinct pathogenic signatures among the JIA subtypes. Gene expression patterns of sJIA samples were clustered using an unsupervised consensus clustering method and the molecular characteristics with clinical significance in the identified clusters were delineated. In particular, molecular signatures and their changes in terms of treatment outcome were systemically explored for a dataset of sJIA patients on IL-1 inhibitors.

## Methods

### Data collection

We searched publications and datasets featuring genes expressed in juvenile idiopathic arthritis (JIA) in Pubmed (https://pubmed.ncbi.nlm.nih.gov/) and Gene Expression Omnibus (https://www.ncbi.nlm.nih.gov/geo/) databases using the following keywords: ‘juvenile idiopathic arthritis,’ ‘Still’s disease,’ ‘transcriptomics,’ ‘microarray,’ and ‘RNA sequencing,’ and finally obtained 15 datasets suitable for analysis with GEO series IDs (GSE7753, GSE11083, GSE13501, GSE13849, GSE15645, GSE20307, GSE21521, GSE26112, GSE41831, GSE55319, GSE58667, GSE67596, GSE79970, GSE80060, and GSE112057). Baseline clinical profiles of each dataset were pooled into Supplementary File 1. For baseline analysis, samples from patients with inactive disease and post-treatment samples were excluded. Duplicated samples with the same ID and patient information obtained from the same institution were also excluded. The combined dataset included blood samples from 168 sJIA, 254 paJIA, 96 oaJIA, and 40 ERA patients as well as 220 healthy controls. The GSE80060 dataset consisted of samples from two randomized trials of canakinumab in sJIA. Patients with active sJIA received subcutaneous canakinumab or placebo and blood samples for ribonucleic acid isolation were collected at baseline (*n* = 104) and at day 3 (*n* = 80). An analysis of the treatment response was compared in samples at baseline and on day 3.

### Preprocess of gene expression data

All analyses were conducted in R (version 4.4.1, The R Project for Statistical Computing, www.r-project.org). In one-channel Affymetrix microarrays, the Robust Multi-array Average (RMA) method installed in the affy package was applied for background correction, normalization, and probe-set summarization [16]. Illumina gene expression array data were preprocessed using the lumi package and normalized by using the robust spline normalization method [17]. Residual technical batch effects occurring from the integration of multiple heterogenous data were adjusted using the ComBat method [18, 19]. Quality assurance and distribution bias were evaluated by principal component analysis. Systematic and dataset-specific bias was significantly diluted after preprocessing as compared with before normalization and batch correction (Supplementary Fig. 1).

### Unsupervised clustering analysis

The Monte Carlo Reference-based Consensus Clustering (M3C) package was used to cluster the samples using the partitioning around medoids (PAM) algorithm and assess the clustering patterns of gene expression profiles for significance [20]. A Monte Carlo simulation generates null distributions of stability scores along the range of K, reject the null hypothesis K = 1 by comparing it with real stability scores, and finally decides the optimum K. The cumulative distribution function (CDF), the proportion of ambiguous clustering (PAC) score, adjusted *P* value, and entropy were used to determine optimum K [21]. The CDF curves show a flat middle segment only for the true K, suggesting that very few sample pairs are ambiguous when K is correctly inferred [22]. PAC score indicated the ambiguity of cluster assignments between clustering runs based on the CDF of the consensus matrix. A low PAC value indicated a flat middle segment. K was more significant at a lower *P* value and entropy. Cluster stability was further verified using the Jaccard similarities index [23]. Generally, a valid, stable cluster should yield a mean Jaccard similarity value of 0.75 or more.

### Filtration of differentially expressed genes

Differentially expressed genes (DEGs) were identified using the limma package, which is based on the empirical Bayes approach [24]. *P* values were adjusted by the Benjamini-Hochberg method, and DEGs were defined as the adjusted *P* value < 0.05 and the absolute value of fold change > 1.5.

### Signaling pathway and biological processes enrichment analysis

Functional enrichment analysis for the upregulated DEGs was performed using Enrichr software, which implements adjusted *P* value, odds ratio and the combined score as the featured enrichment index [25]. Gene-set enrichment analysis (GSEA) was used to identify a set of genes that are over-represented in a given list of genes, compared to a background set of genes [26]. The normalized enrichment score (NES) was used to compare analysis results across the gene-set by accounting for differences in gene-set size and correlations between sets and the expression dataset. Gene-set information on the signaling pathway or biological processes was retrieved from the Kyoto Encyclopedia of Genes and Genomes (KEGG), Gene Ontology (GO), and the Reactome database [27–29]. Single-sample enrichment scores of the gene sets were estimated with gene-set variation analysis (GSVA) using the gsva function in the R package GSVA [30]. Enriched processes were further evaluated by blood transcriptome modular repertoire analysis [31, 32], which includes 382 transcriptome modules based on blood genes co-expression patterns. Information on the modules can be accessed at https://ayllonbe.github.io/modulesV3/index.html. The module response is expressed as the percentage of transcripts constituting a given module showing significant, differential expression between study groups.

### Interferon-stimulated genes scores

The interferon (IFN)-stimulated gene (ISG) score was calculated using the AUCell package [33] and a list of ISGs was retrieved from the Molecular Signatures Database (MSigDB) [26].

### Human protein-protein interaction network and identification of influential nodes

A human protein-protein interaction network was constructed using the Human Interactome Database [34]. Nodes and edges represent proteins and functional or physical interactions between them, respectively, and the network includes 18,853 nodes and 483,037 edges. The connectivity and influence within the network of disease modules were assessed by calculating the local and global network centralities. The spreading score is reflective of the potential of vertices in spreading information within a network and the hubness score is reflective of the sovereignty of a vertex in its surrounding local territory. The integrated value of influence (IVI) combines the local, semi-local and global centralities to unify them in a single influence score [35].

### Molecular signature-based drug screening

The Library of Integrated Network-Based Cellular Signatures (LINCS) is an NIH Common Fund program that catalogs gene expression changes in different types of cells exposed to a variety of perturbing agent that affect normal cellular functions to improve our understanding of normal and diseased cellular states at systems level [36, 37]. This database has been useful for drug repurposing through drug connectivity mapping [38]. We used the metaLINCS R package [39] to perform a meta-level GSEA on the output, which combines them with compounds, based on the ranked correlation scores for each experiment.

### Statistical analysis

For continuous distributed data, between-group comparisons were performed using one-way analysis of variance (ANOVA), paired or unpaired *t*-test. Categorical or dichotomous variables were compared using the chi-squared test or Fisher’s exact test. Correlation analysis between two variables was carried out using Pearson’s method.

## Results

### Differential enrichment of JIA-associated pathways

**Fig. 1:**
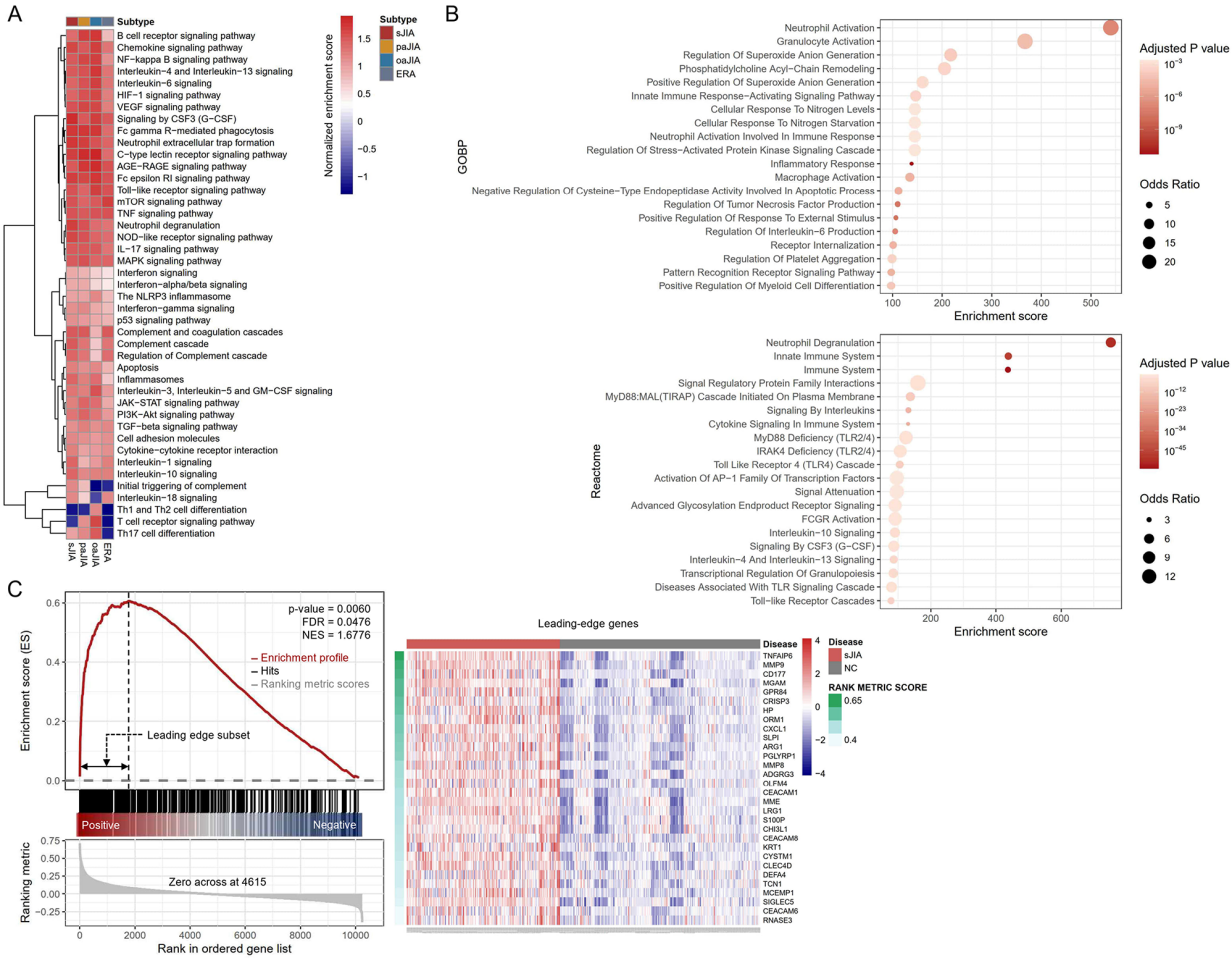
Molecular signatures of sJIA. (**A**) Enrichment of JIA-associated signaling pathways and biological processes by GSEA across the JIA subtypes. Normalized enrichment score (NES) indicates the relative degree of overrepresentation across the gene sets. (**B**) DEG-driven enrichment analysis of sJIA. Upper and lower panels are Gene Ontology (GO) – Biological Processes (BP) and Reactome terms, respectively. (**C**) GSEA plot of the neutrophil degranulation in sJIA. GSEA = gene-set enrichment analysis, sJIA = systemic juvenile idiopathic arthritis, paJIA = polyarticular juvenile idiopathic arthritis, oaJIA = oligoarticular juvenile idiopathic arthritis, ERA = enthesitis-related arthritis

We curated the biological processes and signaling pathways involved in the pathogenesis of JIA from the literature [1, 5, 8, 40]. We then performed a GSEA for the gene expression profiles of each JIA subtype in comparison with healthy controls to understand the differential enrichment of biological processes or pathogenic pathways across the JIA subtypes (Fig. 1A). Major pathways were activated in common across the JIA subtypes, albeit to varying degrees, while some pathways were uniquely activated in specific subtypes. Signaling by CSF3 (G-CSF), Fcγ R-mediated phagocytosis, neutrophil extracellular trap (NET) formation, neutrophil degranulation, and IL-1 signaling pathway was commonly activated and mostly enriched in sJIA. IL-18 signaling pathway was not enriched in oaJIA but B-cell and T-cell receptor signaling pathways and Th17 cell differentiation were enriched more in paJIA and oaJIA.

We performed DEG-driven functional enrichment analysis for the 409 upregulated DEGs of sJIA (Fig. 1B). Neutrophil activation and neutrophil degranulation were the top-ranked enriched processes in Gene Ontology – Biological Process and Reactome panels, respectively. Pathways involving Toll-like receptors (TLRs), Fcγ receptors, G-CSF, and ILs were also detected in the order of priority. In GSEA, TNFAIP6, MMP9, CD177, MGAM, and GPR84 were the top five leading-edge genes in neutrophil degranulation (Fig. 1C).

### Identification of functional molecular subgroups of sJIA

**Fig. 2:**
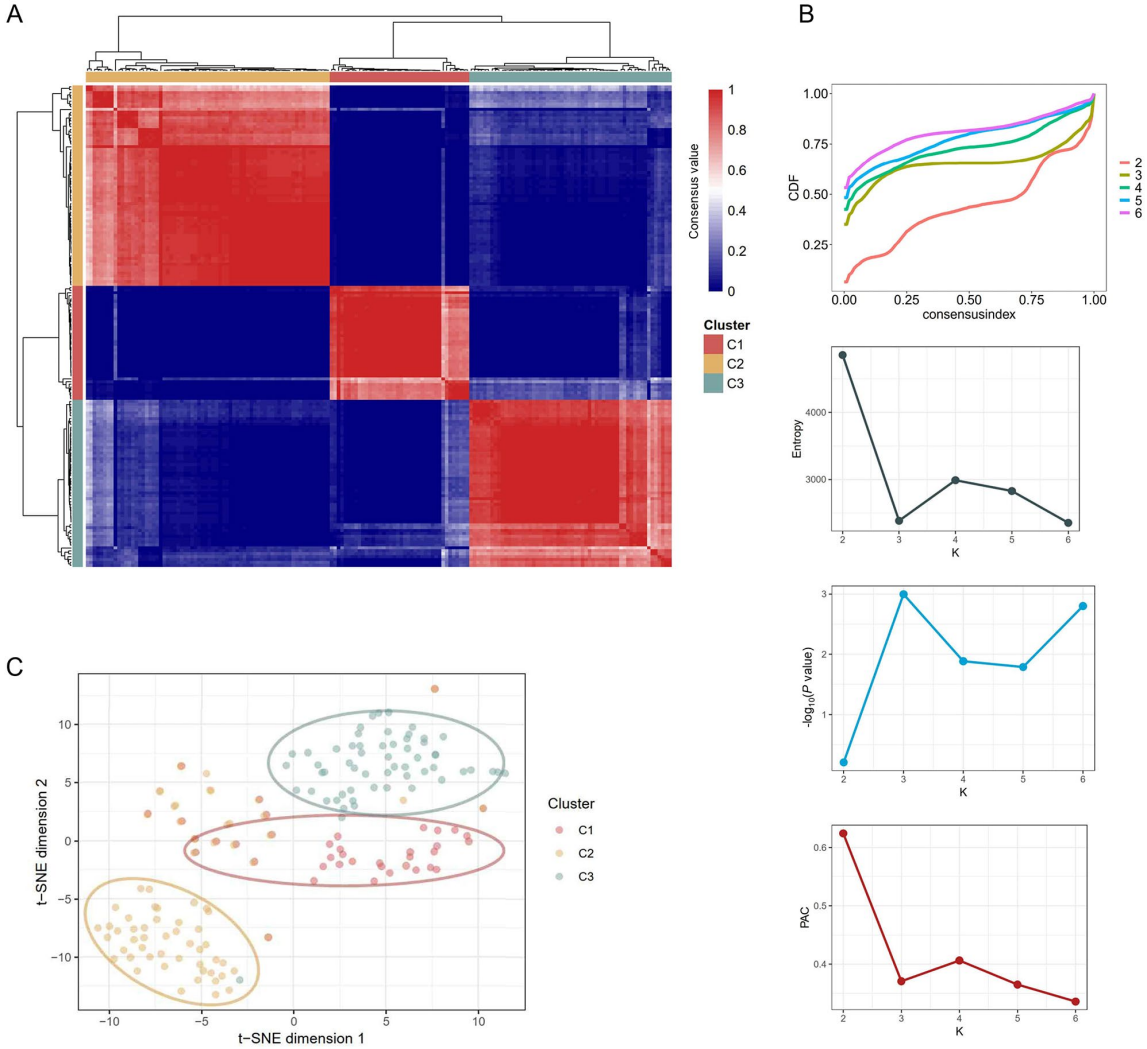
Unsupervised consensus clustering. (**A**) Consensus heatmap of the three subgroups. The subgroups were designated C1(*n* = 40), C2(*n* = 70), and C3(*n* = 58). The consensus value ranges from 0 (never clustered together) to 1 (always clustered together) marked by blue and red colors. (**B**) Determination of the optimum number K of clustering. The cumulative distribution function (CDF), adjusted *P* value, entropy, and proportion of ambiguous clustering (PAC) were used to determine the optimum K of clustering. See the interpretation in the Methods. (**C**) *t*-Distributed stochastic neighbor embedding (*t*-SNE) analysis of the clustered gene expression profiles

To define the molecular subgroups of sJIA, we ran a Monte-Carlo Reference-based Consensus Clustering algorithm for the gene expression profiles of 168 sJIA patients of active and new-onset status. Determination indices suggest that three clusters (designated as C1 [*n* = 40], C2 [*n* = 70], and C3 [*n* = 58]) are the optimum separation (Fig. 2A-B). The segregation of the three subgroups was confirmed by *t*-stochastic neighbor embedding (*t*-SNE) (Fig. 2C). C1 and C3 were adjoined but C2 was relatively isolated. The Jaccard similarity score for C1, C2, and C3 was 0.8803, 0.8649, and 0.7948, respectively, indicating that the identified subgroups were highly stable [23].

### Differential activation of sJIA-associated pathways in three subgroups

**Fig. 3:**
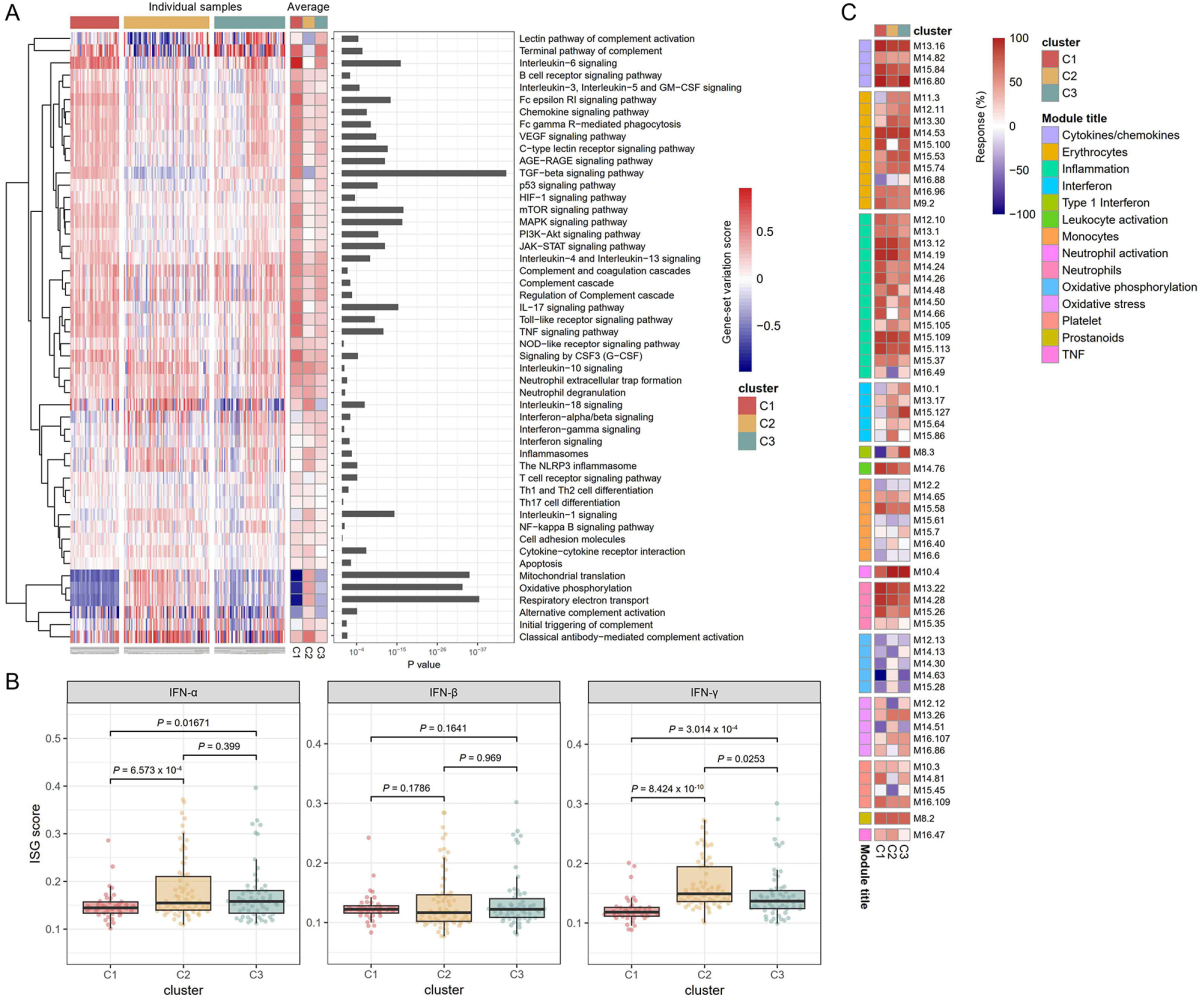
Molecular signatures of the sJIA subgroups. (**A**) Gene-set variation analysis for the JIA-associated signaling pathways and biological processes. Gene-set variation score ranges from − 1 (minimum) to 1 (maximum) marked by blue and red colors. The left, middle, and right panels indicate the enrichment scores at individual sample levels, the average enrichment score of the subgroups, and *P*-values by ANOVA, respectively. (**B**) Interferon-stimulated genes (ISGs) scores. An unpaired *t*-test determined the *P* value. IFN = interferon. (**C**) Blood transcriptome modular repertoire analysis. The module response (%) ranges from − 100 to 100 marked by blue and red. Detailed information on the modules can be accessed at https://ayllonbe.github.io/modulesV3/index.html

DEGs were identified by comparing the gene expression profiles between each subgroup and 220 normal control samples (1,356 genes in C1, 454 genes in C2, and 290 genes in C3), and 145 genes including IL1R1, IL1R2, IL1B, S100A9, and S100A12 were shared between the three subgroups (Supplementary Fig. 2). In DEGs-driven enrichment analysis, neutrophil degranulation was identified to be the most enriched process in all the subgroups (Supplementary Fig. 3). Macrophage activation and TLR-associated signaling pathways were noticeable in C1, while type I interferon (IFN) signaling pathway signal was detected in C2 and C3. GSVA further corroborated the differential activation of the sJIA-associated pathways (Fig. 3A). The IL-1- and IL-18 signaling pathway and inflammasome were activated mostly in C2. The IL-18 signaling pathway was inadequate in C3. Mitochondrial function including mitochondrial translation and oxidative phosphorylation was suppressed in C1 and C3 [41]. IFN-stimulated gene (ISG) scores were significantly higher in C2 and C3 compared with C1 (Fig. 3B). Differential activation in the sJIA-associated pathways between the three subgroups was confirmed by GSEA (Supplementary Fig. 4).

To further characterize the molecular variability of the subgroups, we used the blood transcriptome modular repertoire, which include 382 transcriptome modules based on genes co-expression patterns across 16 diseases and 985 unique transcriptome profiles [31, 32]. The three subgroups showed the different molecular fingerprints at module-level analysis (Fig. 3C and Supplementary Fig. 5). Neutrophil-associated modules (M13.22, M14.28, and M15.26) were upregulated in all subgroups and most significant in C1, whereas the neutrophil activation module (M10.4) was strongest in C3. Modules associated with IL-1, IL-18, and TNF (M15.113, M13.12, and M15.109) were also upregulated across the subgroups. Inflammation (M14.24) and monocyte modules (M14.65, M15.58) were also noticeable. IFN-associated modules (M15.127, M8.3) were activated in C2 and C3 and modules associated with oxidative phosphorylation (M14.30, M14.63, and M15.28) were under-expressed in C1 and C3.

### Expression of biomarkers in three subgroups

**Fig. 4:**
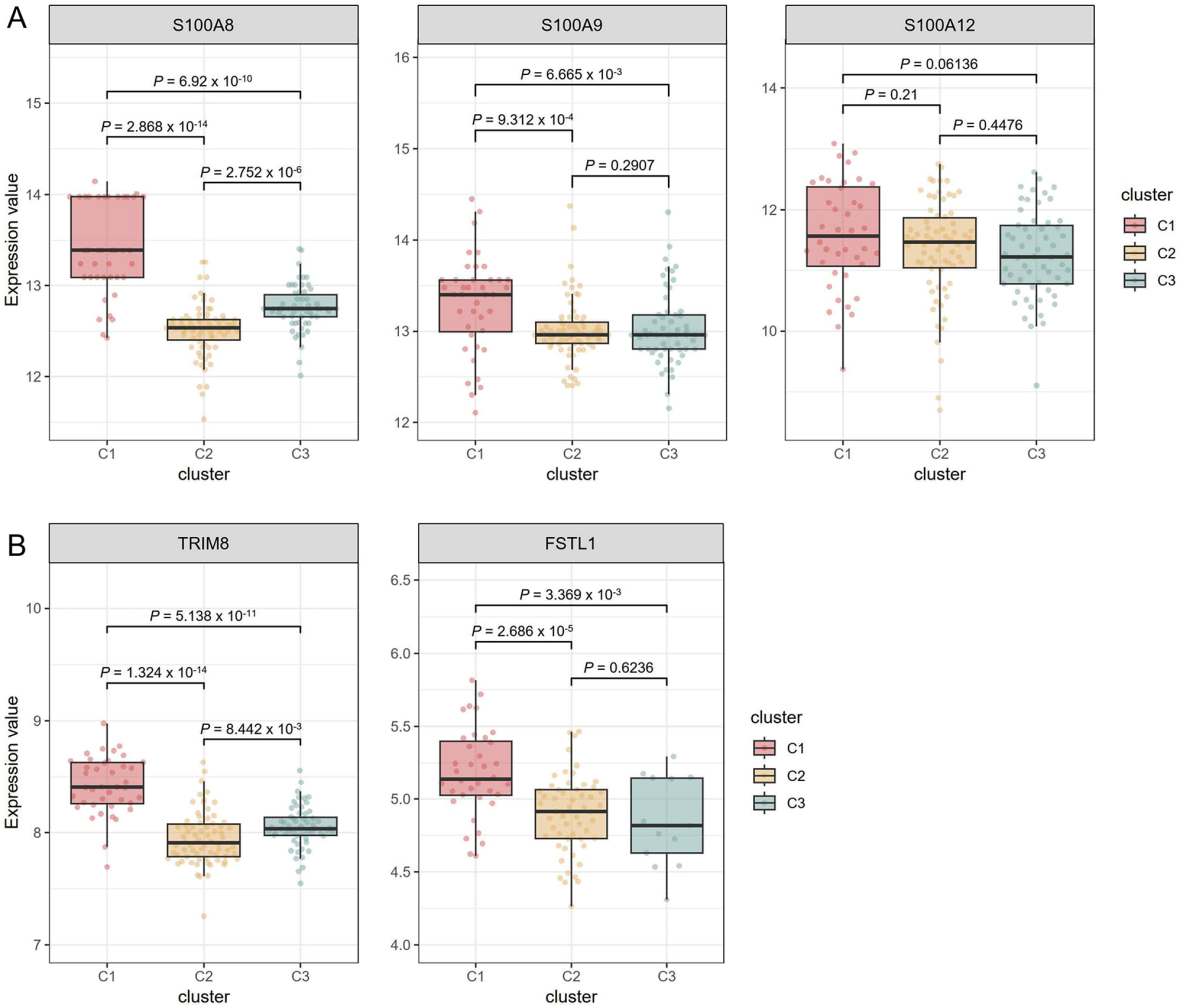
Differential expression of the featured genes. (**A**) Expression of S100 proteins across the sJIA subgroups. (**B**) Expression of MAS-associated genes across the sJIA subgroups. An unpaired *t*-test determined the *P*-value

S100 proteins are the most studied biomarkers that define JIA subtypes, measure disease activity, and predict response to therapy and relapse [42, 43]. S100A8 (FC = 1.54, adjusted *P* value = 3.26 × 10^− 28^), S100A9 (FC = 1.68, adjusted *P* value = 4.38 × 10^− 25^), and S100A12 (FC = 1.86, adjusted *P* value = 1.15 × 10^− 29^) were among the significant DEGs of active sJIA. Expression of S100A8 and S100A9 were highest in C1, while S100A12 expression was comparable across all the subgroups (Fig. 4A). Overexpression of follistatin-like protein 1 (FSTL-1) and tripartite motif containing 8 (TRIM8) was reported to be linked to MAS development [44, 45]. Expression of FSTL-1 and TRIM8 was significantly higher in C1 compared with C2 and C3 (Fig. 4B). FSTL-1 (FC = 1.51, adjusted *P* value = 7.11 × 10^− 11^) and TRIM8 (FC = 1.54, adjusted *P* value = 4.25 × 10^− 30^) were DEGs in C1 but not in C2 and C3.

### Molecular signatures regarding the clinical response to IL-1 inhibitor

**Fig. 5:**
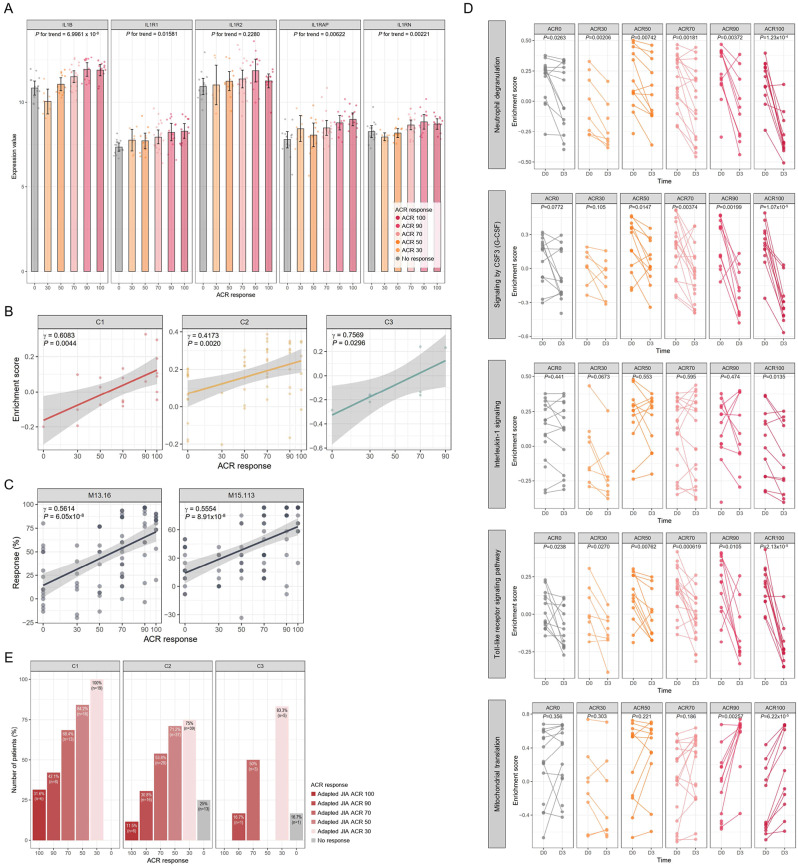
Differential changes in molecular signatures by treatment response to IL-1 inhibitor. (**A**) Expression values of the top 5 leading-edge genes in IL-1 signaling pathways by GSEA. (**B**) Correlation between enrichment score of NF-κB signaling pathway and ACR response. The correlation coefficient and *P* value were obtained by Pearson’s method. (**C**) Correlation between the two most significant modules (M13.16 and M15.113) in blood transcriptome modular repertoire analysis and ACR response. The correlation coefficient and *P* value were obtained by Pearson’s method. (**D**) Temporal changes of the key featured signaling pathways between baseline and day 3 after treatment. A paired *t*-test determined the *P*-value. (**E**) Proportion of ACR response across the three subgroups. The adapted JIA ACR 30, JIA ACR 50, JIA ACR 70, JIA ACR 90, and JIA ACR 100 responses were defined as improvements of at least 30%, 50%, 70%, 90%, and 100%, respectively

A blood transcriptomic dataset, GSE80060, was built from the two randomized trials of canakinumab in sJIA [46]. Patients with active sJIA were to receive subcutaneous canakinumab or placebo and blood samples for RNA isolation were collected at baseline (*n* = 104) and at day 3 (*n* = 80). The IL-1 signaling pathway was one of the enriched processes in sJIA. IL1R1, IL1R2, IL1B, IL1RN, and IL1RAP, were the top 5 leading-edge genes in IL-1 signaling pathway (Supplementary Fig. 6). The ACR response was significantly associated with the expression of IL1B (*P* for trend = 6.9961 × 10^− 8^), which was followed by IL1RN, IL1RAP, and IL1R1 (All *P* for trend < 0.05) (Fig. 5A). ACR response was correlated with the activation of the NF-κB signaling pathway in common for the three subgroups (C1: γ = 0.6083, *P* = 0.0044, C2: γ = 0.4173, *P* = 0.0020, and C3: γ = 0.7569, *P* = 0.0296, respectively) (Fig. 5B). In the blood transcriptome modular repertoire analysis, treatment response was most correlated with M13.16 (γ = 0.5614, *P* = 6.05 × 10^− 8^) and M15.113 (γ = 0.5554, *P* = 8.91 × 10^− 8^), which have close functional ties with neutrophil degranulation and the IL-1 signaling pathway, respectively (Fig. 5C).

Changes from baseline to day 3 in the major enriched pathways and dysfunctional processes by ACR response were examined. Activated pathways, such as neutrophil degranulation, NET formation, signaling by CSF3 (G-CSF), Fcγ R-mediated phagocytosis, and the TLR signaling pathway, at baseline were effectively subdued at day 3 as the treatment response improved (Fig. 5D and Supplementary Fig. 7). Suppression of the IL-1 signaling pathway was observed only in the ACR100 group (*P* = 0.0135) and the scale of reduction was not as remarkable as in other pathways. Mitochondrial functions such as mitochondrial translation and oxidative phosphorylation were significantly recovered in the ACR90 and ACR100 groups (Fig. 5D and Supplementary Fig. 7). Analysis of high response modules in the blood transcriptome modular repertoire reproduced similar results. However, the response of M10.4 (neutrophil activation) was not effectively controlled and even increased in the ACR100 group unlike other neutrophil-associated modules (M13.22, M14.28, and M15.26) (Supplementary Fig. 7).

Next, we further examined the molecular signatures in the three subgroups. The treatment response of patients receiving canakinumab in the three subgroups is depicted in Fig. 5E. All patients in C1 showed a response over ACR30, but 25% (*n* = 13) of C2 and 16.7% (*n* = 1) of C3 showed no response.

### Identification of key influential genes in the disease module

**Fig. 6:**
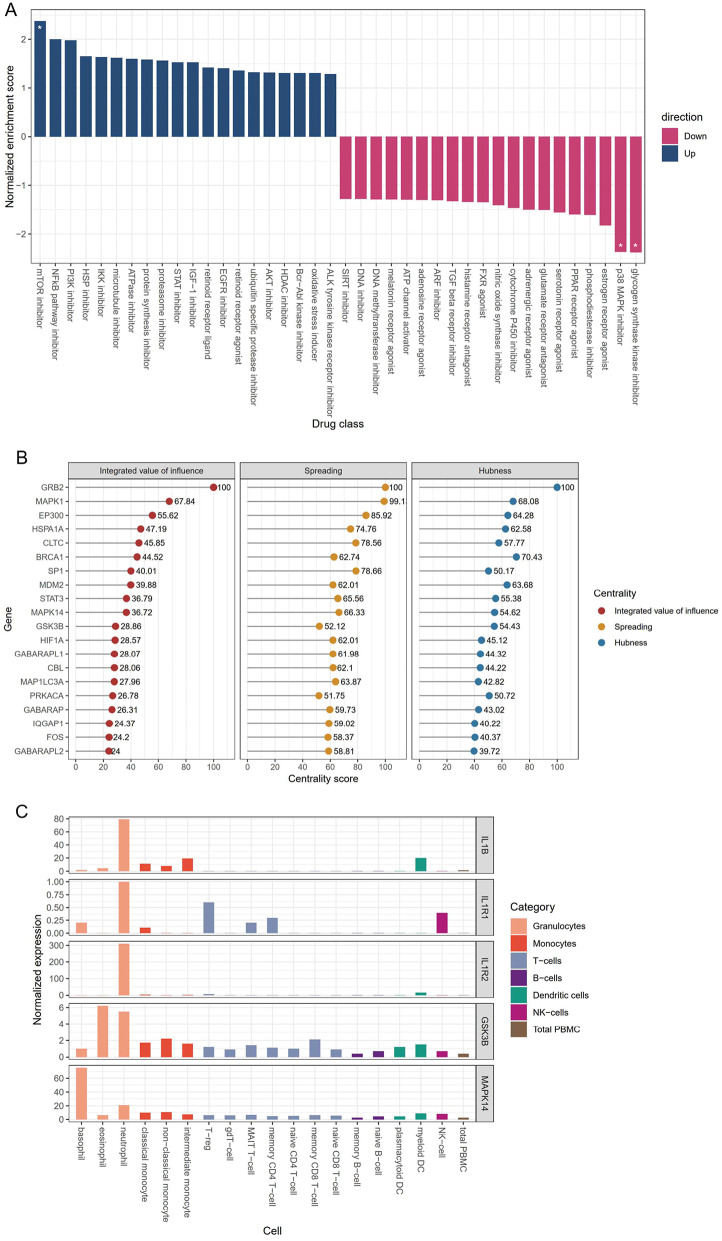
Identification of key influential genes with therapeutic potentials. (**A**) The enriched compound classes that agree or oppose the C1 gene expression signatures and their enrichment score. A negative normalized enrichment score indicates that a compound could counteract a given gene expression profiles. Asterisks indicate statistical significance (*P* value < 0.05). (**B**) The integrated value of influence, spreading, and hubness of the genes in the C1 molecular network of disease module. The spreading score reflects the ability of the node to propagate information and the hubness score measures the impact of each node within its domain. The integrated value of influence is the synergistic product of hubness and spreading values. (**C**) Expression values of the IL1B, IL1R1, IL1R2, GSK3B, and MAPK14 in the immune cell subsets. The data was sourced from the Human Protein Atlas (https://www.proteinatlas.org/)

To discover potential drugs or drug targets with promising therapeutic effects, we used novel approach to evaluate the correlation between sJIA gene expression profiles and perturbation signatures from the LINCS L1000 database using the metaLINCS package [39]. A negative normalized enrichment score (NES) indicates that a compound could counteract a gene expression profile. Glycogen synthetase kinase (GSK) inhibitor and p38 MAPK (also known as MAPK14) inhibitor were the drug classes that significantly counteract the gene expression of the C1 and C3 subgroups (Fig. 6A and Supplementary Fig. 8). GSK3B is an element of M14.24 (response = 89.4%), and MAPK14 is an element of M15.84 (response = 90.9%) and M15.113 (response = 90%). These modules were designated as cytokines/chemokines and inflammation modules. No chemical drug class was found to significantly antagonize the gene expression of C2 in the current library of the LINCS database.

We calculated the integrated value of influence (IVI) for each gene in a DEG-driven network. IVI summarizes numerous network parameters, such as hubness and degree centrality, to provide a metric of the overall importance of each gene in a network [35]. MAPK14 and GSK3B were ranked highly as influential nodes in the network (Fig. 6B). MAPK1, MDM2, and STAT3 were also found to be significant influential nodes. The expression levels of the genes in the immune cell subsets were examined using the human protein atlas database [47]. IL1B, IL1R1, IL1R2, and GSK3B in neutrophils were expressed at a distinctly higher value compared with other immune cells and MAPK14 was also highly expressed in neutrophils (Fig. 6C).

## Discussion

In the present study, we made a comprehensive gene expression profile of the blood from patients with JIA including sJIA, paJIA, oaJIA, and ERA, and examined the variation in pathogenic features across the JIA subtypes. In particular, novel molecular subgroups of sJIA were identified based on their blood molecular signatures by an advanced unsupervised clustering method and their shared and distinct molecular characteristics were explored. Neutrophil activation/degranulation and activation of the IL-1 signaling pathway were key processes that the three subgroups had in common. Proinflammatory signals, including TNF, IL-6, TLR, and G-SCF signaling pathways, were also identified with variation across the subgroups, and C1 was the most inflammatory subset. The type I IFN signature, IL-18 signaling pathway, mitochondrial dysfunction, and MAS risk profile were the notable features distinguishing the subgroups. In a canakinumab-treated dataset, treatment response was correlated with IL1B expression and NF-κB signaling pathway at baseline, and neutrophil-associated processes were effectively suppressed in a good responder group. GSK3B and p38 MAPK inhibition was a promising alternative strategy for counteracting the perturbed gene expression of sJIA.

Neutrophils are the most abundant leukocytes in the circulation and first responders to acute inflammation [48]. sJIA is characterized by neutrophilia on laboratory examination and was significantly enriched for neutrophil degranulation and the IL-1 signaling pathway. G-CSF signaling, Fcγ R-medicated phagocytosis, NET formation were also activated. G-CSF is responsible for neutrophilia and extramedullary myelopoiesis [49]. The stimulation of Fcγ receptors is one of the robust signals that induce NET formation via MAPK signaling pathway [50, 51]. High concentration of gasdermin D was reported in Still’s disease [52]. Gasdermin D is not only critically involved in the IL-1β secretion, macrophage activation, and NLRP3 inflammation activation [52–54] but also plays a vital role in the NET formation [55]. In contrast, paJIA and oaJIA were enriched in B- and T-cell receptor signaling and Th17 cell differentiation, indicating they have more autoimmune characteristics. In ERA, distinct inflammatory responses associated with enthesitis or arthritis may be localized and not fully captured in blood gene signatures, with the exception of the IL-18 signaling pathway.

The clinical variation and biological heterogeneity of sJIA are major hurdles to reach a satisfactory outcomes [8, 56]. There have been experiences where modular approaches or molecular stratification in inflammatory diseases, such as systemic lupus erythematosus, Sjögren’s syndrome, and rheumatoid arthritis, provided deeper clinical and mechanistic insights into the disease subtypes and have also introduced interesting therapeutic points of view [10–15]. We identified three subgroups of sJIA with distinct molecular signatures. Proinflammatory signals such as IL-6, TNF, TLR, JAK-STAT, and MAPK signaling pathways were most activated in the C1 subgroup, which was followed by the C3 subgroup. However, IL-1 and IL-18 signaling pathways and inflammasome were enriched more in C2 subgroup. IFN-stimulated signatures were detected at higher levels in C2 and C3. Innate and adaptive immune response-related processes or autoinflammatory and autoimmune characteristics were mixed and each subgroup cannot be clearly defined as innately adaptive or truly autoimmune according to disease progression [8]. S100A8 or S100A9 are released from the IL-1β -activated monocytes or neutrophils and their serum levels are an important biomarker to predict response to therapy and risk for relapse [5, 43]. The C1 subgroup had the most inflammatory characteristics, had a high expression of S100A8 and S100A9, and showed a better response to canakinumab. Thus, the C1 subgroup might show a favorable response to TNF inhibitors or IL-6 inhibitors as alternative treatment options. It is also intriguing that the expression of TRIM8 and FSTL1 in the C1 subgroup was significantly higher compared with that in the C2 and C3 subgroups. TRIM8 augments macrophage responses to IFN-γ, the pivotal cytokine in MAS [45], and FSTL1 enhances IL-1β and IL-6 secretion from monocytes or macrophages and induces macrophage proliferation [57]. A signature of monocyte or macrophage activation was enriched in C1 and if the patients of C1 subgroup are primed by IFN-γ, they might be at a high risk of developing MAS. The C2 subgroup is unique with strong IL-1 and IL-18 signals but weak IL-6 and TNF signals and preserved mitochondrial function. C3 subgroup has moderate inflammatory properties and is similar to C1 except for an upregulated IFN signature.

IL-1β is transduced via IL-1Rs. In particular, IL-1R1 binds to IL-1β with higher affinity than IL-1R2. The complex IL-1β/IL-1R1 results in a conformational change of the receptor that allows the binding with IL-1RAP, a second receptor subunit, and finally triggers a signaling cascade resulting in the activation of the NF-κB and MAPK pathways [58, 59]. IL-1-targeted therapy is one of the mainstays in the treatment of sJIA and canakinumab is a specific inhibitor of IL-1β [7]. In the canakinumab-treated dataset, patients showed a better response to canakinumb as the expression levels of IL1B, IL1R1, and IL1RAP were higher but not IL1R2. However, the activity of the IL-1 signaling pathway itself was not suppressed in correlation with the treatment response except for the ACR100 responder group. Instead, IL-1-directed cellular processes, such as neutrophil degranulation, NET formation, G-CSF signaling pathway, Fcγ R-mediated phagocytosis, and the TLR signaling pathway, were effectively suppressed as the treatment response improved. The treatment response might be dependent on the suppression of neutrophil-oriented proinflammatory processes via the inhibition of IL-1 signaling pathways rather than IL-1 inhibition itself. Module M10.4, associated with neutrophil activation and enriched for the defense response to bacterium, was highly active in a considerable portion of patients at baseline but not controlled at all even with canakinumab treatment. It is proposed that primed neutrophils do not completely turn off their inherent engine even under clinically complete response and primed to reignite. It was reported that neutrophils from sJIA patients with long-standing chronic inactive disease status demonstrated elevated inflammatory gene expression, including inflammasome components and S100A8 [60].

A feed-forward loop involving IL-1β and S100 proteins between monocytes and neutrophils is a key module contributing to the perpetuation of chronic inflammation in sJIA [5, 8]. An understanding of the limitations of biologic therapy against a single pathogenic cytokine was obtained from experience with rheumatoid arthritis [61] and the same is true for sJIA [3, 4]. Interventions affecting IL-1β activity over time could interrupt the proinflammatory cycle but might not be enough to induce complete remission and prevent relapse with DMARD discontinuation. We identified two novel intracellular molecular targets with the potentials to counteract the dysregulated gene expression of sJIA. GSK3β is a serine/threonine kinase with a broad array of cellular targets, such as cytoskeletal proteins and transcription factors. GSK3β is a constitutively active kinase that is regulated by phosphorylation. In various sterile inflammatory models such as collagen-induced arthritis, ischemia-reperfusion injury, streptozotocin-induced diabetes, and Alzheimer’s disease, GSK3β activation was induced and administration of GSK3β inhibitors effectively reduced inflammatory response [62]. For instance, in a collagen-induced model of arthritis, treatment with a GSK3 inhibitor reduced joint inflammation and leukocyte infiltration and decreased the production of proinflammatory cytokine such as IL-1β, TNF, IL-6, and IFN-γ [63]. GSK3β inhibitors effectively reduce the production of the proinflammatory cytokines IL-1β, IFN-γ, and IL-6 in TLR-stimulated peripheral blood mononuclear cells by differentially regulating NF-κB and CREB [64]. An essential role of the p38α (MAPK14) pathway in inflammatory responses and inflammatory diseases including rheumatoid arthritis and chronic obstructive pulmonary disease is well-established [65, 66]. TLR stimulation of neutrophils results in activation of the p38 MAPK pathway, ultimately regulating NF-κB activation and enhanced expression of the TNF gene [67]. However, reports suggest that GSK3β and p38 MAPK negatively regulate each other by indirect intervention or direct phosphorylation in specific cell lines [68, 69]. A better understanding of how the GSK3 and p38 MAPK interact, work in cooperation, and affect the inflammatory activity of neutrophils in sJIA is needed. GSK3β and p38 MAPK are highly expressed in neutrophils among blood immune cells, but GSK3β and p38 MAPK inhibitors would not be readily applicable because of the adverse effects arising from the global inhibition of kinases, especially in pediatric patients. However, this result is important in the sense that it provides an intriguing mechanistic insight into the hub molecules operating the disease module and the opportunity to discover promising therapeutic targets in sJIA.

There are several limitations to be addressed. First, there are deficits in the integrity of the gene expression profiles. Some genes, albeit possibly minor, were missed during the integration of multiple datasets with different list of sequencing probes. Additionally, the correction method of batch effect might not be ideal. Second, we did not fully examine the association between molecular subgroups and clinical manifestations (fever, rash, arthritis, etc.), laboratory variables (ESR and CRP) and long-term disease course or outcome due to lack of information at an individual level. Third, blood molecular signatures can be driven by the predominant blood cell subset, probably neutrophils. Neutrophilia is a signature finding in the blood of active sJIA. However, distinct key molecular signatures were captured depending on the nature of the disease [10–15]. Furthermore, a broader single-cell study would be needed to assess single-cell variability. Fourth, blood molecular signatures could not fully reflect the root immunologic abnormalities in the peripheral immune organs (spleen, thymus and lymph node) or target organ (arthritic joints). Fifth, due to the limited sample size, the datasets could not be effectively split, and the three-cluster scheme should be validated using an independent dataset.

Enormous progress has been made concerning the treatment of sJIA and a significant outcome was achieved by using anti-cytokine biologic agents. However, questions, including optimal selection of initial therapy, adequate maintenance targets, and personalized medicine based on biomarkers, remain to be addressed [7]. The initial step is the rational stratification of the patients based on molecular signatures. In this study, we identified three subgroups of sJIA with distinct molecular signatures and a different balance of cytokine patterns. Whether each molecular subgroup has its own unique trait for an individual subject or switches to other subgroups over the disease course need to be investigated. It is noteworthy that the activity of neutrophils is the key indicator of the inflammatory response and was significantly associated with treatment response. Furthermore, we narrowed the window of novel therapeutic targets by presenting the hub molecules that direct key inflammatory modules in activated neutrophils. Future research on the detailed clinical characteristics of the molecular subgroups may be useful for clinical trial design or treatment selection.

## Electronic supplementary material

Below is the link to the electronic supplementary material.

Supplementary Material 1: Supplementary File 1

Supplementary Material 2: Supplementary Figures

## Data Availability

No datasets were generated or analysed during the current study.

## Abbreviations

ANOVA: One-way analysis of variance
CDF: Cumulative distribution function
DEGs: Differentially expressed genes
DMARDs: Disease-modifying antirheumatic drugs
ERA: Enthesitis-related arthritis
FSTL-1: Follistatin-like protein 1
GO: Gene Ontology
GSEA: Gene-set enrichment analysis
GSK: Glycogen synthetase kinase
GSVA: Gene-set variation analysis
IFN: Interferon
IL: Interleukin
ISG: Interferon-stimulated gene
JIA: Juvenile idiopathic arthritis
KEGG: KYOTO Encyclopedia of Genes and Genomes
LINCS: THE Library of Integrated Network-Based Cellular Signatures
MAS: Macrophage activation syndrome
M3C: MONTE Carlo Reference-based Consensus Clustering
MSigDB: Molecular Signatures Database
NES: Normalized enrichment score
NET: Neutrophil extracellular trap
NSAIDs: Non-steroidal anti-inflammatory drugs
oaJIA: Oligoarticular juvenile idiopathic arthritis
PAC: The proportion of ambiguous clustering
paJIA: Polyarticular juvenile idiopathic arthritis
sJIA: Systemic juvenile idiopathic arthritis
TLRs: Toll-like receptors
TNF: Tumor necrosis factor
TRIM8: Tripartite motif containing 8
t-SNE: T-stochastic neighbor embedding

## References

[1] Martini A, Lovell DJ, Albani S, Brunner HI, Hyrich KL, Thompson SD, et al. Juvenile idiopathic arthritis. Nat Rev Dis Primers. 2022;8:5. 35087087 10.1038/s41572-021-00332-8

[2] Young Dae K, Alan VJ, Woojin C. Differential diagnosis of Juvenile Idiopathic Arthritis. J Rheumatic Dis. 2017;24:131–7.

[3] Ambler WG, Nanda K, Onel KB, Shenoi S. Refractory systemic onset juvenile idiopathic arthritis: current challenges and future perspectives. Ann Med. 2022;54:1839–50. 35786149 10.1080/07853890.2022.2095431 PMC9258439

[4] Brunner HI, Schanberg LE, Kimura Y, Dennos A, Co DO, Colbert RA, et al. New medications are needed for children with juvenile idiopathic arthritis. Arthritis Rheumatol. 2020;72:1945–51. 32524767 10.1002/art.41390 PMC7722045

[5] Mellins ED, Macaubas C, Grom AA. Pathogenesis of systemic juvenile idiopathic arthritis: some answers, more questions. Nat Rev Rheumatol. 2011;7:416–26. 21647204 10.1038/nrrheum.2011.68 PMC4180659

[6] Petty RE, Laxer RM, Lindsley CB, Wedderburn L, Fuhlbrigge RC, Mellins ED. Textbook of Pediatric Rheumatology E-Book. Elsevier Health Sciences; 2020.

[7] Hinze CH, Foell D, Kessel C. Treatment of systemic juvenile idiopathic arthritis. Nat Rev Rheumatol. 2023;19:778–89. 37923864 10.1038/s41584-023-01042-z

[8] Kessel C, Hedrich CM, Foell D. Innately adaptive or truly autoimmune: is there something Unique about systemic juvenile idiopathic arthritis? Arthritis Rheumatol. 2020;72:210–9. 31524322 10.1002/art.41107

[9] Gattorno M, Piccini A, Lasigliè D, Tassi S, Brisca G, Carta S, et al. The pattern of response to anti-interleukin-1 treatment distinguishes two subsets of patients with systemic-onset juvenile idiopathic arthritis. Arthritis Rheum. 2008;58:1505–15. 18438814 10.1002/art.23437

[10] Chaussabel D, Quinn C, Shen J, Patel P, Glaser C, Baldwin N, et al. A modular analysis framework for blood genomics studies: application to systemic lupus erythematosus. Immunity. 2008;29:150–64. 18631455 10.1016/j.immuni.2008.05.012 PMC2727981

[11] Chiche L, Jourde-Chiche N, Whalen E, Presnell S, Gersuk V, Dang K, et al. Modular transcriptional repertoire analyses of adults with systemic lupus erythematosus reveal distinct type I and type II interferon signatures. Arthritis Rheumatol. 2014;66:1583–95. 24644022 10.1002/art.38628 PMC4157826

[12] Jourde-Chiche N, Whalen E, Gondouin B, Speake C, Gersuk V, Dussol B, et al. Modular transcriptional repertoire analyses identify a blood neutrophil signature as a candidate biomarker for lupus nephritis. Rheumatology (Oxford). 2017;56:477–87. 28031441 10.1093/rheumatology/kew439

[13] Soret P, Le Dantec C, Desvaux E, Foulquier N, Chassagnol B, Hubert S, et al. A new molecular classification to drive precision treatment strategies in primary Sjögren’s syndrome. Nat Commun. 2021;12:3523. 34112769 10.1038/s41467-021-23472-7 PMC8192578

[14] James JA, Guthridge JM, Chen H, Lu R, Bourn RL, Bean K, et al. Unique Sjögren’s syndrome patient subsets defined by molecular features. Rheumatology (Oxford). 2020;59:860–8. 31497844 10.1093/rheumatology/kez335 PMC7188221

[15] Tasaki S, Suzuki K, Kassai Y, Takeshita M, Murota A, Kondo Y, et al. Multi-omics monitoring of drug response in rheumatoid arthritis in pursuit of molecular remission. Nat Commun. 2018;9:2755. 30013029 10.1038/s41467-018-05044-4 PMC6048065

[16] Gautier L, Cope L, Bolstad BM, Irizarry RA. Affy–analysis of Affymetrix GeneChip data at the probe level. Bioinformatics. 2004;20:307–15. 14960456 10.1093/bioinformatics/btg405

[17] Du P, Kibbe WA, Lin SM. Lumi: a pipeline for processing Illumina microarray. Bioinformatics. 2008;24:1547–8. 18467348 10.1093/bioinformatics/btn224

[18] Johnson WE, Li C, Rabinovic A. Adjusting batch effects in microarray expression data using empirical Bayes methods. Biostatistics. 2007;8:118–27. 16632515 10.1093/biostatistics/kxj037

[19] Zhang Y, Parmigiani G, Johnson WE. ComBat-seq: batch effect adjustment for RNA-seq count data. NAR Genom Bioinform. 2020;2:lqaa078. 33015620 10.1093/nargab/lqaa078 PMC7518324

[20] John CR, Watson D, Russ D, Goldmann K, Ehrenstein M, Pitzalis C, et al. M3C: Monte Carlo reference-based consensus clustering. Sci Rep. 2020;10:1816. 32020004 10.1038/s41598-020-58766-1 PMC7000518

[21] Șenbabaoğlu Y, Michailidis G, Li JZ. Critical limitations of consensus clustering in class discovery. Sci Rep. 2014;4:6207. 25158761 10.1038/srep06207 PMC4145288

[22] Wilkerson MD, Hayes DN. ConsensusClusterPlus: a class discovery tool with confidence assessments and item tracking. Bioinformatics. 2010;26:1572–3. 20427518 10.1093/bioinformatics/btq170 PMC2881355

[23] Hennig C. Cluster-wise assessment of cluster stability. Comput Stat Data Anal. 2007;52:258–71.

[24] Ritchie ME, Phipson B, Wu D, Hu Y, Law CW, Shi W, et al. Limma powers differential expression analyses for RNA-sequencing and microarray studies. Nucleic Acids Res. 2015;43:e47. 25605792 10.1093/nar/gkv007 PMC4402510

[25] Kuleshov MV, Jones MR, Rouillard AD, Fernandez NF, Duan Q, Wang Z, et al. Enrichr: a comprehensive gene set enrichment analysis web server 2016 update. Nucleic Acids Res. 2016;44:W90–7. 27141961 10.1093/nar/gkw377 PMC4987924

[26] Subramanian A, Tamayo P, Mootha VK, Mukherjee S, Ebert BL, Gillette MA, et al. Gene set enrichment analysis: a knowledge-based approach for interpreting genome-wide expression profiles. Proc Natl Acad Sci U S A. 2005;102:15545–50. 16199517 10.1073/pnas.0506580102 PMC1239896

[27] Kanehisa M, Furumichi M, Tanabe M, Sato Y, Morishima K. KEGG: new perspectives on genomes, pathways, diseases and drugs. Nucleic Acids Res. 2017;45:D353–61. 27899662 10.1093/nar/gkw1092 PMC5210567

[28] Ashburner M, Ball CA, Blake JA, Botstein D, Butler H, Cherry JM, et al. Gene ontology: tool for the unification of biology. The Gene Ontology Consortium. Nat Genet. 2000;25:25–9. 10802651 10.1038/75556 PMC3037419

[29] Milacic M, Beavers D, Conley P, Gong C, Gillespie M, Griss J, et al. The Reactome Pathway Knowledgebase 2024. Nucleic Acids Res. 2024;52:D672–8. 37941124 10.1093/nar/gkad1025 PMC10767911

[30] Hänzelmann S, Castelo R, Guinney J. GSVA: gene set variation analysis for microarray and RNA-seq data. BMC Bioinformatics. 2013;14:7. 23323831 10.1186/1471-2105-14-7 PMC3618321

[31] Altman MC, Rinchai D, Baldwin N, Toufiq M, Whalen E, Garand M, et al. Development of a fixed module repertoire for the analysis and interpretation of blood transcriptome data. Nat Commun. 2021;12:4385. 34282143 10.1038/s41467-021-24584-w PMC8289976

[32] Rinchai D, Roelands J, Toufiq M, Hendrickx W, Altman MC, Bedognetti D, et al. BloodGen3Module: blood transcriptional module repertoire analysis and visualization using R. Bioinformatics. 2021;37:2382–9. 33624743 10.1093/bioinformatics/btab121 PMC8388021

[33] Deng Y, Zheng Y, Li D, Hong Q, Zhang M, Li Q, et al. Expression characteristics of interferon-stimulated genes and possible regulatory mechanisms in lupus patients using transcriptomics analyses. EBioMedicine. 2021;70:103477. 34284174 10.1016/j.ebiom.2021.103477 PMC8318865

[34] Guthrie J, Köstel Bal S, Lombardo SD, Müller F, Sin C, Hütter CVR, et al. AutoCore: a network-based definition of the core module of human autoimmunity and autoinflammation. Sci Adv. 2023;9:eadg6375. 37656781 10.1126/sciadv.adg6375 PMC10848965

[35] Salavaty A, Ramialison M, Currie P. Integrated Value of Influence: an integrative method for the identification of the most influential nodes within networks. Patterns (N Y). 2020;1:100052. 33205118 10.1016/j.patter.2020.100052 PMC7660386

[36] Keenan AB, Jenkins SL, Jagodnik KM, Koplev S, He E, Torre D, et al. The Library of Integrated Network-Based Cellular Signatures NIH Program: System-Level cataloging of human cells response to perturbations. Cell Syst. 2018;6:13–24. 29199020 10.1016/j.cels.2017.11.001 PMC5799026

[37] Koleti A, Terryn R, Stathias V, Chung C, Cooper DJ, Turner JP, et al. Data portal for the Library of Integrated Network-based Cellular Signatures (LINCS) program: integrated access to diverse large-scale cellular perturbation response data. Nucleic Acids Res. 2018;46:D558–66. 29140462 10.1093/nar/gkx1063 PMC5753343

[38] Musa A, Ghoraie LS, Zhang SD, Glazko G, Yli-Harja O, Dehmer M, et al. A review of connectivity map and computational approaches in pharmacogenomics. Brief Bioinform. 2018;19:506–23. 28069634 10.1093/bib/bbw112 PMC5952941

[39] Kwee I, Martinelli A, Khayal LA, Akhmedov M. metaLINCS: an R package for meta-level analysis of LINCS L1000 drug signatures using stratified connectivity mapping. Bioinform Adv. 2022;2:vbac064. 36699415 10.1093/bioadv/vbac064 PMC9710587

[40] Zaripova LN, Midgley A, Christmas SE, Beresford MW, Baildam EM, Oldershaw RA. Juvenile idiopathic arthritis: from aetiopathogenesis to therapeutic approaches. Pediatr Rheumatol Online J. 2021;19:135. 34425842 10.1186/s12969-021-00629-8 PMC8383464

[41] Ishikawa S, Mima T, Aoki C, Yoshio-Hoshino N, Adachi Y, Imagawa T, et al. Abnormal expression of the genes involved in cytokine networks and mitochondrial function in systemic juvenile idiopathic arthritis identified by DNA microarray analysis. Ann Rheum Dis. 2009;68:264–72. 18388159 10.1136/ard.2007.079533

[42] Gohar F, Kessel C, Lavric M, Holzinger D, Foell D. Review of biomarkers in systemic juvenile idiopathic arthritis: helpful tools or just playing tricks? Arthritis Res Ther. 2016;18:163. 27411444 10.1186/s13075-016-1069-z PMC4944486

[43] Ahn JG. Role of biomarkers in Juvenile Idiopathic Arthritis. J Rheumatic Dis. 2020;27:233–40.

[44] Gorelik M, Fall N, Altaye M, Barnes MG, Thompson SD, Grom AA, et al. Follistatin-like protein 1 and the ferritin/erythrocyte sedimentation rate ratio are potential biomarkers for dysregulated gene expression and macrophage activation syndrome in systemic juvenile idiopathic arthritis. J Rheumatol. 2013;40:1191–9. 23678162 10.3899/jrheum.121131 PMC3885333

[45] Schulert GS, Pickering AV, Do T, Dhakal S, Fall N, Schnell D, et al. Monocyte and bone marrow macrophage transcriptional phenotypes in systemic juvenile idiopathic arthritis reveal TRIM8 as a mediator of IFN-γ hyper-responsiveness and risk for macrophage activation syndrome. Ann Rheum Dis. 2021;80:617–25. 33277241 10.1136/annrheumdis-2020-217470

[46] Brachat AH, Grom AA, Wulffraat N, Brunner HI, Quartier P, Brik R, et al. Early changes in gene expression and inflammatory proteins in systemic juvenile idiopathic arthritis patients on canakinumab therapy. Arthritis Res Ther. 2017;19:13. 28115015 10.1186/s13075-016-1212-x PMC5260050

[47] Uhlen MA-O, Karlsson MA-O, Zhong WA-O, Tebani AA-O, Pou CA-OX, Mikes JA-O et al. A genome-wide transcriptomic analysis of protein-coding genes in human blood cells. LID - eaax9198 [pii] LID– 10.1126/science.aax9198 [doi]. 10.1126/science.aax9198 31857451

[48] Herrero-Cervera A, Soehnlein O, Kenne E. Neutrophils in chronic inflammatory diseases. Cell Mol Immunol. 2022;19:177–91. 35039631 10.1038/s41423-021-00832-3 PMC8803838

[49] Malengier-Devlies B, Bernaerts E, Ahmadzadeh K, Filtjens J, Vandenhaute J, Boeckx B, et al. Role for Granulocyte colony-stimulating factor in Neutrophilic Extramedullary myelopoiesis in a murine model of systemic juvenile idiopathic arthritis. Arthritis Rheumatol. 2022;74:1257–70. 35243819 10.1002/art.42104

[50] Chen T, Li Y, Sun R, Hu H, Liu Y, Herrmann M, et al. Receptor-mediated NETosis on neutrophils. Front Immunol. 2021;12:775267. 34804066 10.3389/fimmu.2021.775267 PMC8600110

[51] Kim JW, Ahn MH, Jung JY, Suh CH, Kim HA. An Update on the Pathogenic Role of Neutrophils in Systemic Juvenile Idiopathic Arthritis and Adult-Onset Still’s Disease. Int J Mol Sci. 2021;22. 10.3390/ijms222313038 PMC8657670 34884842

[52] Tang S, Yang C, Li S, Ding Y, Zhu D, Ying S, et al. Genetic and pharmacological targeting of GSDMD ameliorates systemic inflammation in macrophage activation syndrome. J Autoimmun. 2022;133:102929. 36326513 10.1016/j.jaut.2022.102929

[53] Evavold CL, Ruan J, Tan Y, Xia S, Wu H, Kagan JC. The pore-forming protein gasdermin D regulates Interleukin-1 secretion from living macrophages. Immunity. 2018;48:35–e446. 29195811 10.1016/j.immuni.2017.11.013 PMC5773350

[54] Wang C, Yang T, Xiao J, Xu C, Alippe Y, Sun K, et al. NLRP3 inflammasome activation triggers gasdermin D-independent inflammation. Sci Immunol. 2021;6:eabj3859. 34678046 10.1126/sciimmunol.abj3859 PMC8780201

[55] Sollberger G, Choidas A, Burn GL, Habenberger P, Di Lucrezia R, Kordes S et al. Gasdermin D plays a vital role in the generation of neutrophil extracellular traps. Sci Immunol. 2018;3. 10.1126/sciimmunol.aar6689 30143555

[56] Eng SW, Duong TT, Rosenberg AM, Morris Q, Yeung RS. The biologic basis of clinical heterogeneity in juvenile idiopathic arthritis. Arthritis Rheumatol. 2014;66:3463–75. 25200124 10.1002/art.38875 PMC4282094

[57] Mattiotti A, Prakash S, Barnett P, van den Hoff MJB. Follistatin-like 1 in development and human diseases. Cell Mol Life Sci. 2018;75:2339–54. 29594389 10.1007/s00018-018-2805-0 PMC5986856

[58] Mantovani A, Dinarello CA, Molgora M, Garlanda C. Interleukin-1 and related cytokines in the regulation of inflammation and immunity. Immunity. 2019;50:778–95. 30995499 10.1016/j.immuni.2019.03.012 PMC7174020

[59] Dinarello CA. The IL-1 family of cytokines and receptors in rheumatic diseases. Nat Rev Rheumatol. 2019;15:612–32. 31515542 10.1038/s41584-019-0277-8

[60] Brown RA, Henderlight M, Do T, Yasin S, Grom AA, DeLay M, et al. Neutrophils from children with systemic juvenile idiopathic arthritis exhibit Persistent Proinflammatory Activation despite Long-Standing clinically inactive disease. Front Immunol. 2018;9:2995. 30619348 10.3389/fimmu.2018.02995 PMC6305285

[61] Kim M, Choe Y-h, Lee S-i. Lessons from the success and failure of targeted drugs for rheumatoid arthritis: perspectives for Effective Basic and Translational Research. Immune Netw. 2022;22. 10.4110/in.2022.22.e8 PMC8901706 35291656

[62] Hoffmeister L, Diekmann M, Brand K, Huber R. GSK3: a kinase balancing Promotion and Resolution of inflammation. Cells. 2020;9. 10.3390/cells9040820 PMC7226814 32231133

[63] Kwon YJ, Yoon CH, Lee SW, Park YB, Lee SK, Park MC. Inhibition of glycogen synthase kinase-3β suppresses inflammatory responses in rheumatoid arthritis fibroblast-like synoviocytes and collagen-induced arthritis. Joint Bone Spine. 2014;81:240–6. 24176738 10.1016/j.jbspin.2013.09.006

[64] Martin M, Rehani K, Jope RS, Michalek SM. Toll-like receptor-mediated cytokine production is differentially regulated by glycogen synthase kinase 3. Nat Immunol. 2005;6:777–84. 16007092 10.1038/ni1221 PMC1933525

[65] Canovas B, Nebreda AR. Diversity and versatility of p38 kinase signalling in health and disease. Nat Rev Mol Cell Biol. 2021;22:346–66. 33504982 10.1038/s41580-020-00322-w PMC7838852

[66] Gupta J, Nebreda AR. Roles of p38α mitogen-activated protein kinase in mouse models of inflammatory diseases and cancer. Febs j. 2015;282:1841–57. 25728574 10.1111/febs.13250 PMC5006851

[67] Nick JA, Avdi NJ, Young SK, Lehman LA, McDonald PP, Frasch SC, et al. Selective activation and functional significance of p38alpha mitogen-activated protein kinase in lipopolysaccharide-stimulated neutrophils. J Clin Invest. 1999;103:851–8. 10079106 10.1172/JCI5257 PMC408145

[68] Thornton TM, Pedraza-Alva G, Deng B, Wood CD, Aronshtam A, Clements JL, et al. Phosphorylation by p38 MAPK as an alternative pathway for GSK3beta inactivation. Science. 2008;320:667–70. 18451303 10.1126/science.1156037 PMC2597039

[69] Abell AN, Granger DA, Johnson GL. MEKK4 stimulation of p38 and JNK activity is negatively regulated by GSK3beta. J Biol Chem. 2007;282:30476–84. 17726008 10.1074/jbc.M705783200

