# Supplementary material for: Blood molecular subtypes to guide precision treatment strategies in systemic juvenile idiopathic arthritis
Source: Arthritis Res Ther. 2025 Feb 8;27:27. doi: 10.1186/s13075-025-03498-8 (PMC11806610; doi:10.1186/s13075-025-03498-8)
Supplement: Supplementary file 2 — Supplementary Material 2: Supplementary Figures [file 13075_2025_3498_MOESM2_ESM.docx]

**Blood molecular subtypes to guide precision treatment strategies in systemic juvenile idiopathic arthritis**

In-Woon Baek^1^, Jung Woo Rhim^2^, Kyung-Su Park^3^, Ki-Jo Kim^3*^

^1^ Division of Rheumatology, Department of Internal Medicine, Ewha Womans University, College of Medicine, Seoul, Republic of Korea

^2^ Department of Pediatrics, Daejeon St. Mary’s Hospital, College of Medicine, The Catholic University of Korea, Seoul, Republic of Korea

^3^ Division of Rheumatology, Department of Internal Medicine, St. Vincent’s Hospital, College of Medicine, The Catholic University of Korea, Seoul, Republic of Korea

* Correspondence: md21c@catholic.ac.kr (K-J.K.)

**SUPPLEMENTARY FIGURES**


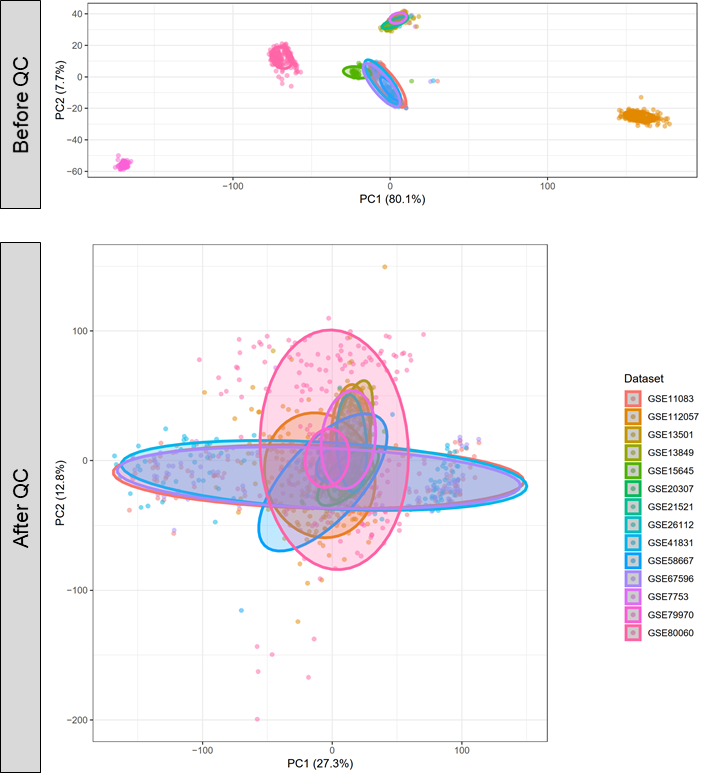


**Figure 1.** Principal component analysis on the integrated datasets of blood transcriptomics before (A) and after (B) normalization and batch correction. QC=quality control.


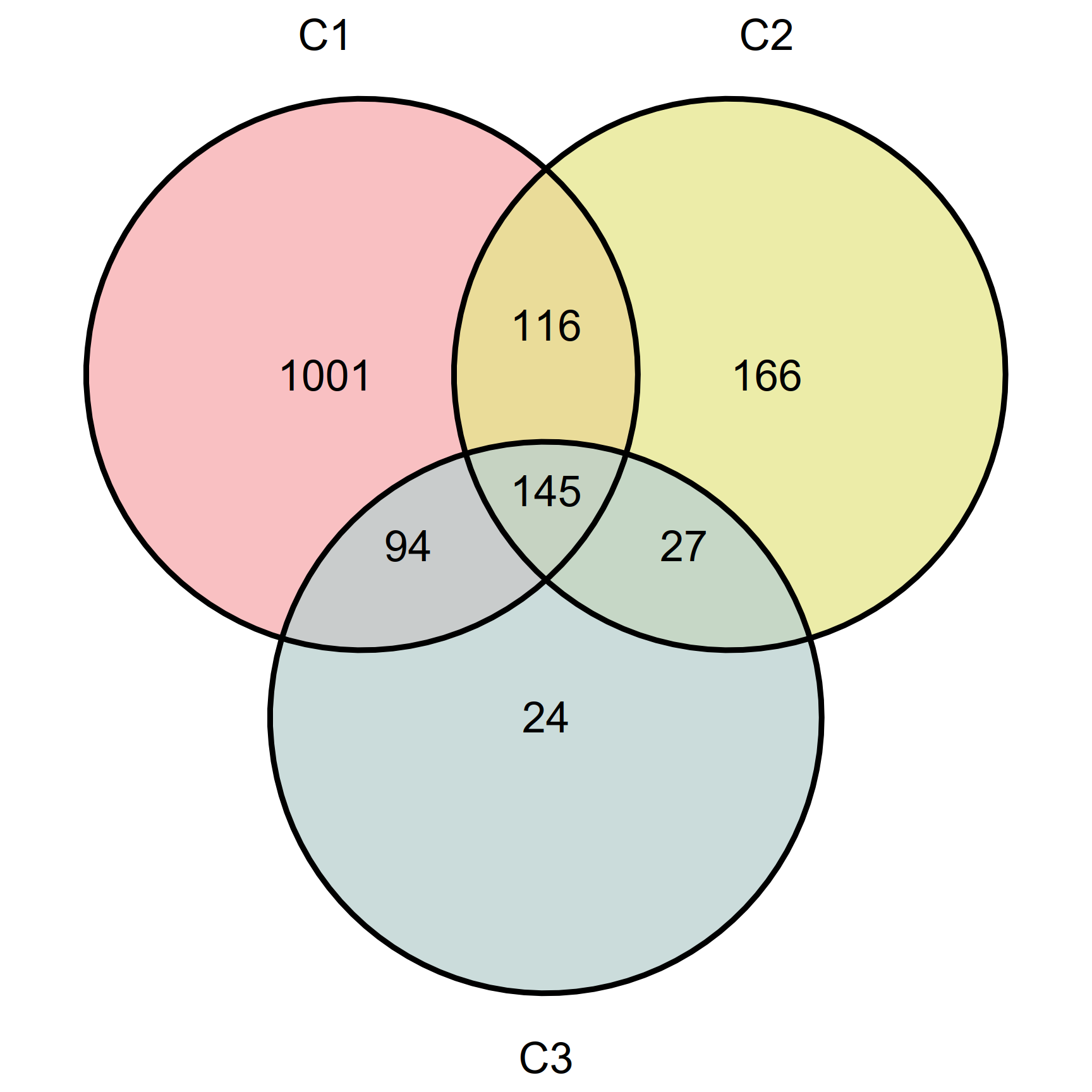


**Figure 2.** Venn diagram of differentially expressed genes (DEGs).


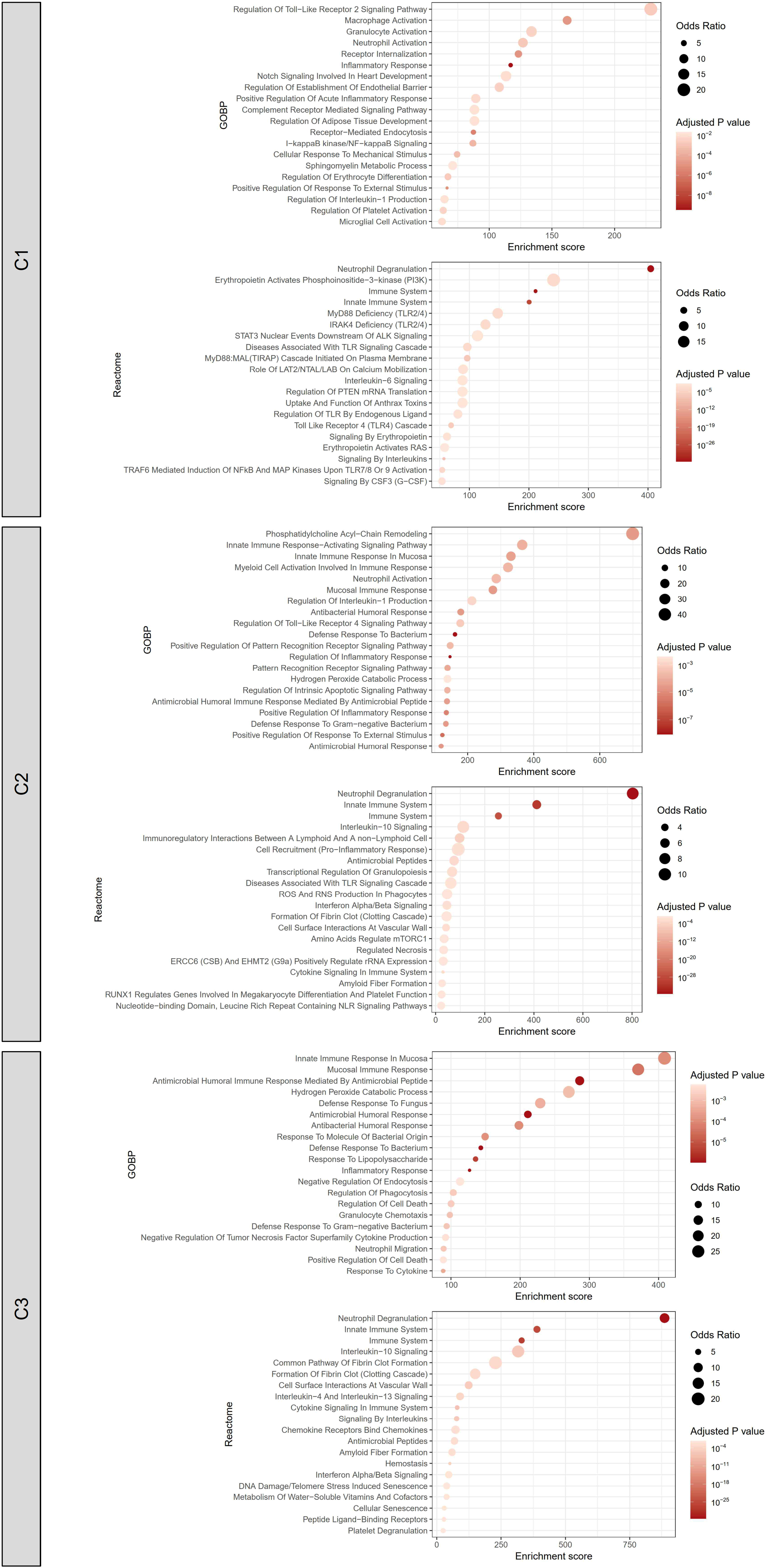


**Figure 3.** Functional enrichment profiles in terms of Gene Ontology (GO) – Biological Process (BP) and Reactome pathways by the DEGs of C1, C2, and C3 subgroups. Enrichment was measured by Fisher’s exact test using the enrichR package.


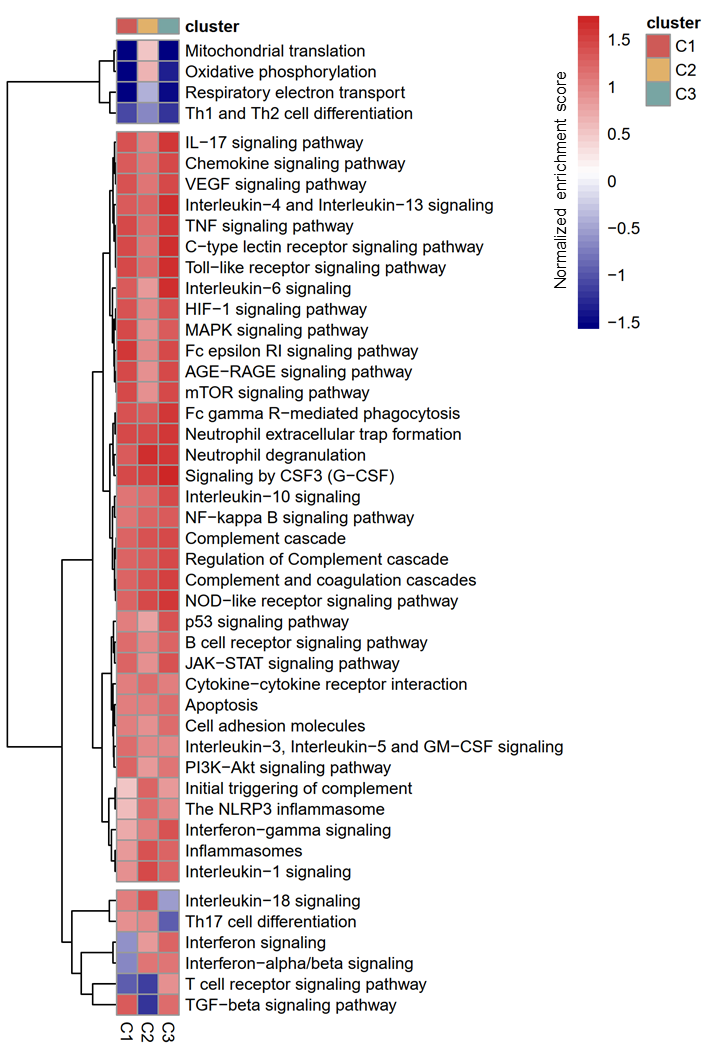


**Figure 4.** Normalized enrichment score (NES) of the JIA-associated signaling pathways and biological processes across the three sJIA subgroups by gene-set enrichment analysis (GSEA).


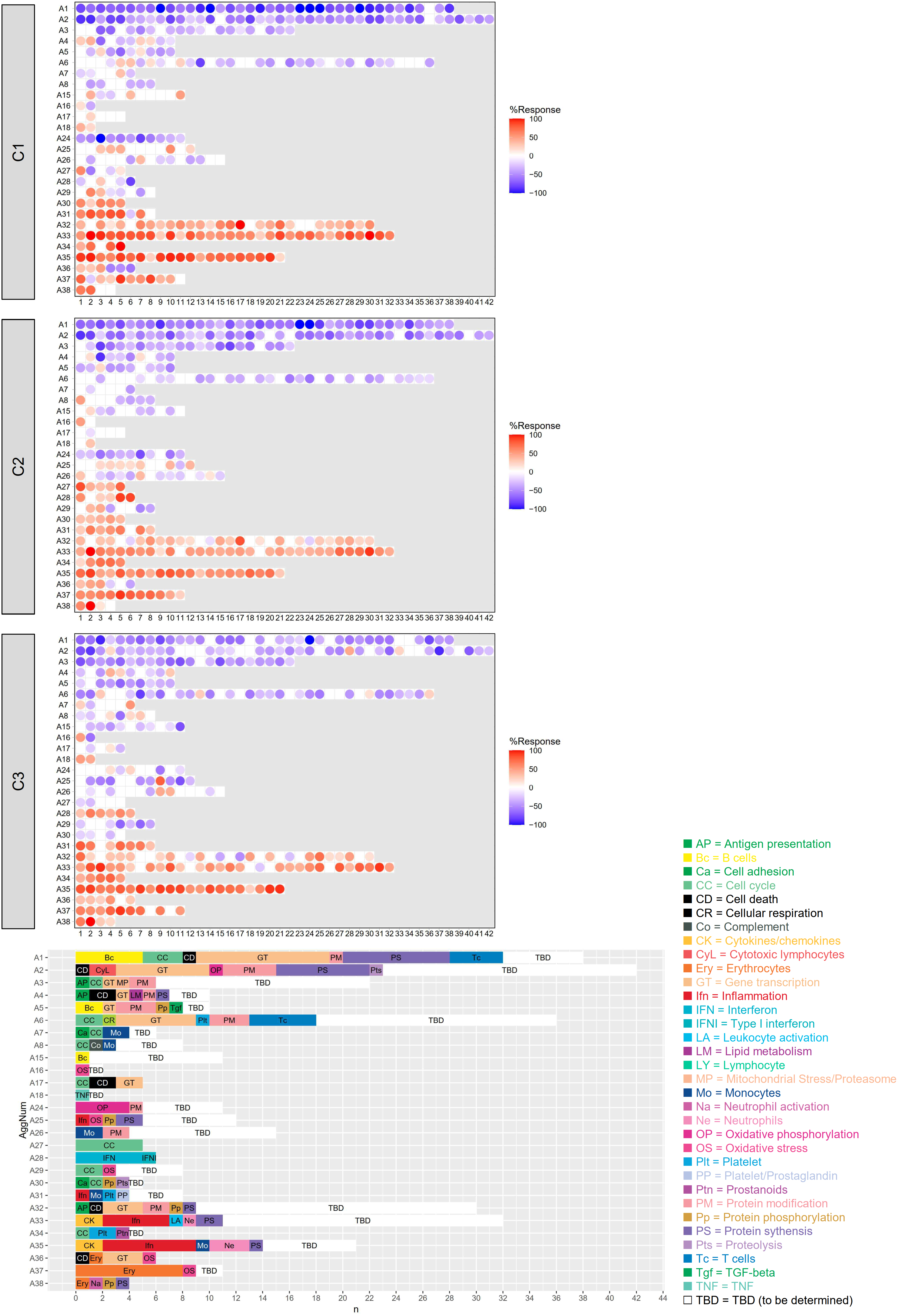


**Figure 5.** Blood transcriptome modular repertoire analysis. The fingerprint grid plot of the three sJIA subgroups. The position of the modules on the grid is fixed and annotated as in the lowest panel. The percent response of individual modules is represented on the grid by red and blue spots of varying color intensity denoting a predominant increase or decrease in abundance, respectively.


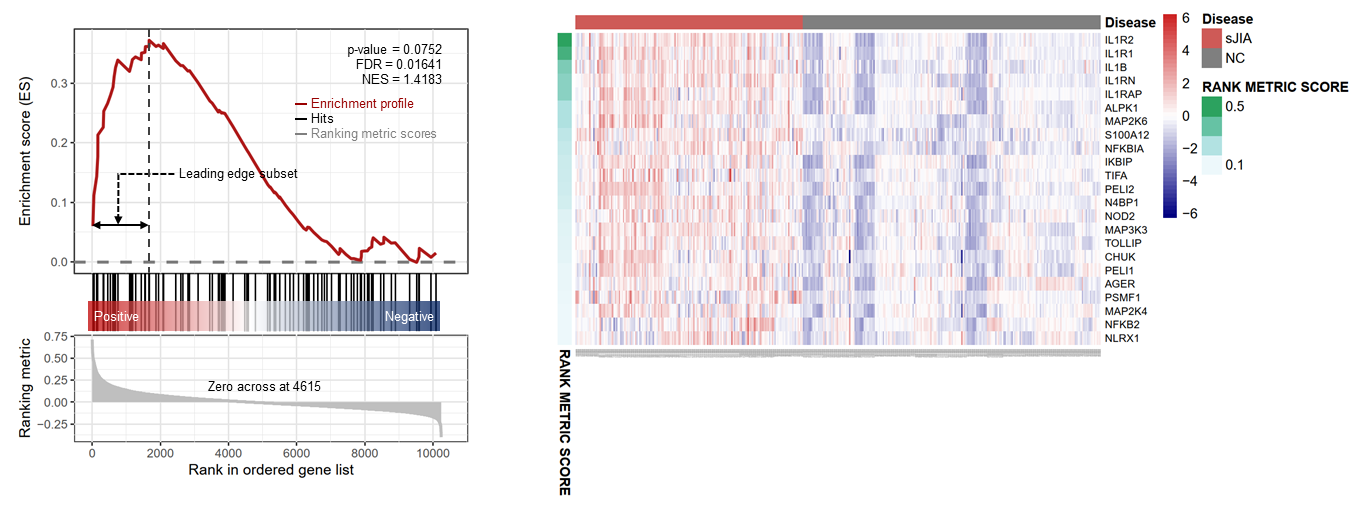


**Figure 6.** Gene-set enrichment analysis (GSEA) of the IL-1 signaling pathway. (Left panel) GSEA plot. (Right panel) the leading-edge genes. sJIA=systemic juvenile idiopathic arthritis, NC=normal control.


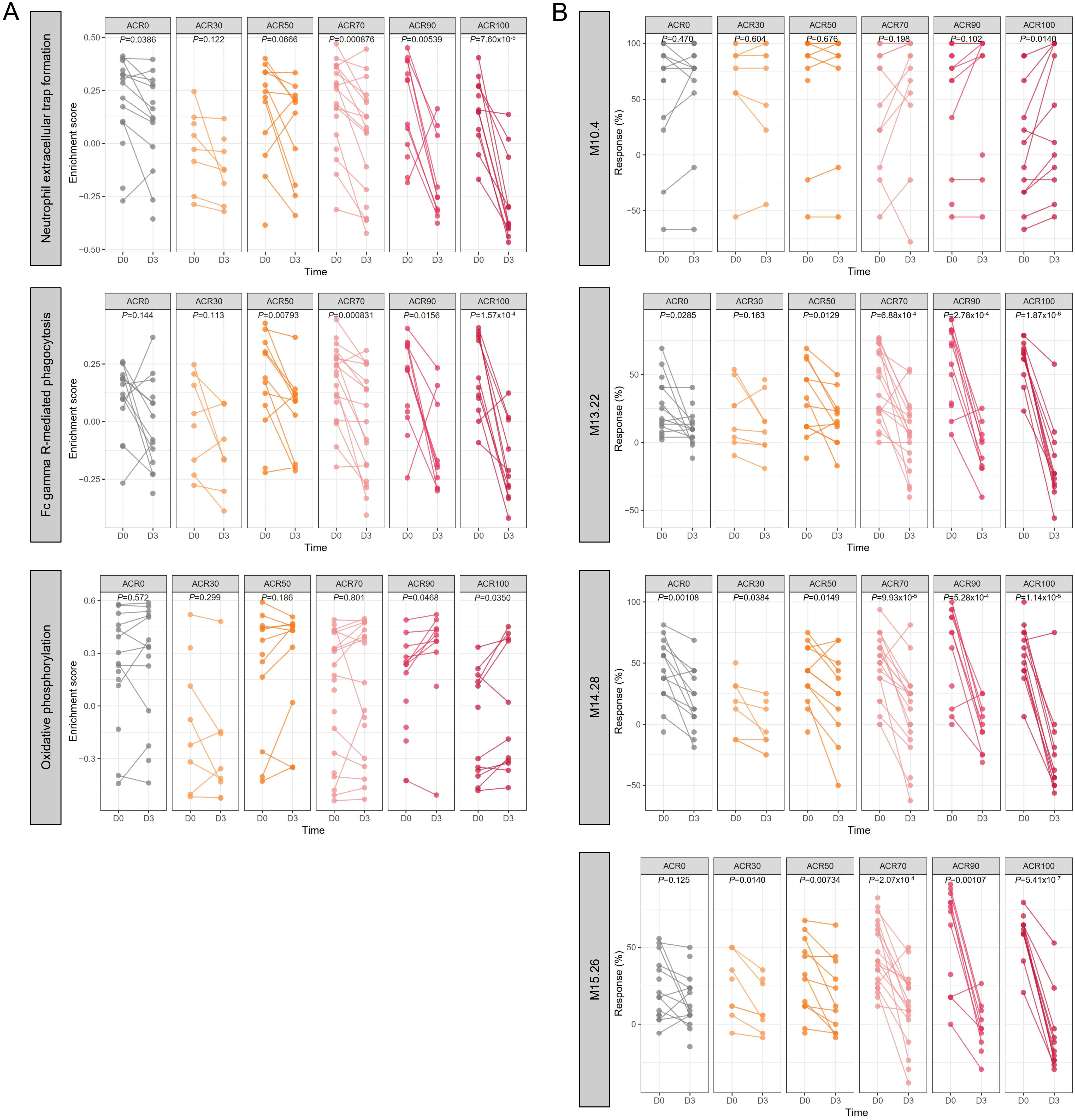


**Figure 7**. (**A**) Temporal changes of the key featured signaling pathways between baseline and day 3 after treatment. (**B**) Temporal changes of the key featured modules between baseline and day 3 after treatment. A paired *t*-test determined the *P*-value.


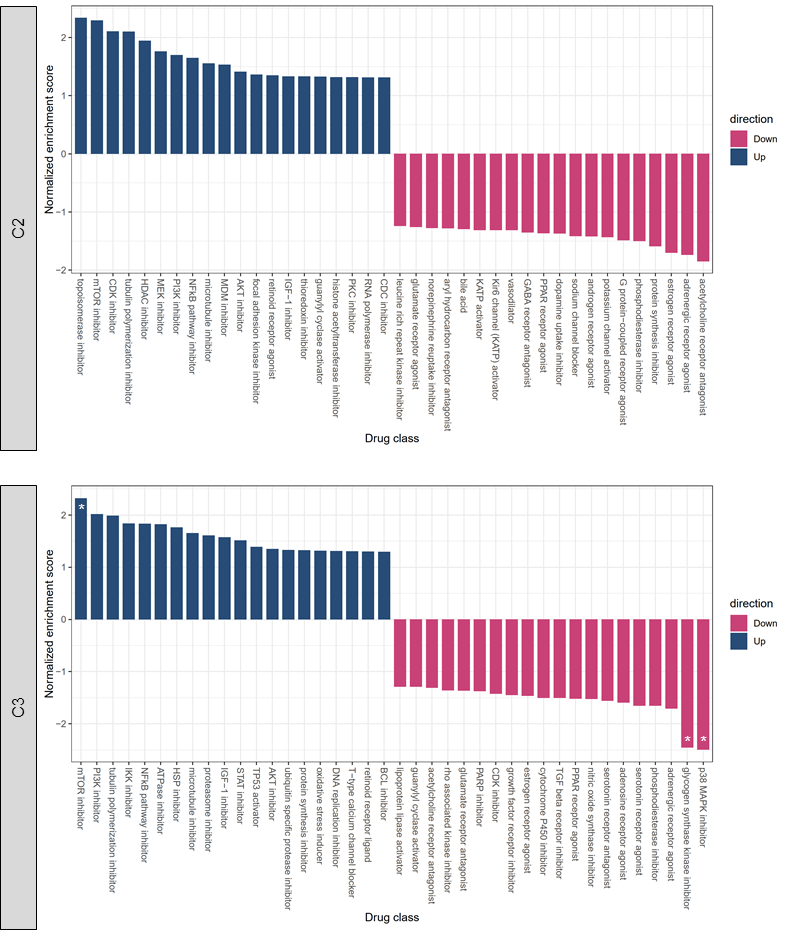


**Figure 8**. Identification of key influential genes with therapeutic potentials. The enriched compound classes agreeing or opposing the C2 (upper panel) and C3 (lower panel) gene expression signatures and their enrichment score. A negative normalized enrichment score indicates that a compound could counteract a given gene expression profiles.
